# Perioperative Care of Patients with Inflammatory Bowel Disease: Focus on Nutritional Support

**DOI:** 10.1155/2018/7890161

**Published:** 2018-09-23

**Authors:** Patrick L. Stoner, Amir Kamel, Fares Ayoub, Sanda Tan, Atif Iqbal, Sarah C. Glover, Ellen M. Zimmermann

**Affiliations:** ^1^Department of Medicine, Division of Gastroenterology, University of Florida, 2000 SW Archer Rd, Gainesville, FL 32610, USA; ^2^Department of Pharmacotherapy, College of Pharmacy, University of Florida, 2000 SW Archer Rd, Gainesville, FL 32610, USA; ^3^Department of Surgery, Division of Colorectal Surgery, College of Medicine, University of Florida, 2000 SW Archer Rd, Gainesville, FL 32610, USA

## Abstract

Patients with inflammatory bowel disease (IBD) commonly require surgery despite the availability of an increasingly large repertoire of powerful immunosuppressive medications for the treatment of IBD. Optimizing patients' care preoperatively is crucial to obtaining good surgical outcomes. This review discusses preoperative assessment and management principles including assessing disease location and activity with cross-sectional or endoscopic imaging, addressing modifiable risk factors (i.e., stopping smoking, weaning steroids, and correcting anemia), and properly managing medications. The major focus of our literature review is the evaluation for malnutrition, a common finding that affects up to 70% of patients with IBD and a well-known, independent risk factor for adverse postoperative outcomes. Our review confirms that whenever feasible, oral or enteral nutrition (EN) is the preferred method of nutritional support; parenteral nutrition (PN) should be reserved for nutritionally deficient IBD patients unable to tolerate EN. In selected patients, recent data demonstrated that the use of preoperative PN resulted in improved nutritional status, fewer postoperative complications, and reduced disease severity. Our review highlights the need for well-designed, prospective trials investigating perioperative nutritional support in patients with IBD. Future studies should perform modern nutritional assessment, standardize for diet, and include patients with UC since this subset of patients is underrepresented in existing studies. In addition, relevant outcome of interest specific to Crohn's disease (CD) patients such as length of small bowel resected, number of anastomoses, and need for an ostomy should be included as these patients may require repeated small bowel resections.

## 1. Introduction

Inflammatory bowel disease (IBD), including Crohn's disease (CD) and ulcerative colitis (UC), is characterized by chronic relapsing gastrointestinal (GI) inflammation. Though the underlying cause of IBD remains unknown, important insights into the pathogenesis have been gained through studies of immune mechanisms, genetics, and the microbiome [1]. In addition, an explosion of new therapeutics has revolutionized our approach to treatment. While new therapies have helped decrease hospital admission rates, incidence rates for surgery remain high. Indeed, the risk of surgery in patients with Crohn's disease is approximately 50% at a 10-year disease duration [2], while 40% of patients with ulcerative colitis requiring inpatient care will ultimately require proctocolectomy [3]. Postoperative endoscopic recurrence of CD is near 90% at one year. In ulcerative colitis, a disease that has historically been considered curative with surgery, significant rates of postoperative transition to CD, difficult to control pouchitis, and systemic inflammatory manifestations remind us that the disease has an immunologic basis that persists even after proctocolectomy [3].

An explanation for the persistently high surgical rates in CD even in the era of aggressive use of potent anti-inflammatory medications eludes the field. The answer can likely be found in the molecular mechanisms that underlie the progression from inflammation to fibrosis causing end organ dysfunction and structural damage such as strictures that often require surgery. Pathogenically, it seems that inflammatory mechanisms trigger fibrotic pathways that march on despite our potent therapeutics. In certain patients, aggressive disease appears to predispose patients to multiple surgeries such that 35% of patients requiring one resection will require a second resection within 10 years [2].

Identifying patients destined to have aggressive IBD can help clinicians tailor therapy and set a strategy for monitoring disease progression. Discussing these risks with patients can help improve patient understanding of their disease and improve their adherence to medication and testing regimens. Understanding the consequences can help patients modify risk factors such as smoking in patients with CD. Predictors of aggressive CD are shown in Table 1 [4–7].

Preoperative assessment and management are key to good outcomes in IBD. Imaging is fundamental to the planning of both medical and surgical strategies as it highlights the sites and severity of inflammation and identifies complications. Colonoscopy has been the primary modality for recognition of dysplasia in IBD, especially ulcerative colitis as the inflamed sites are within the reach of the colonoscope. In CD, ileocolonic disease can be assessed by colonoscopy. In patients with more proximal disease, cross-sectional imaging such as computed tomography enterography (CTE) and magnetic resonance enterography (MRE) can evaluate bowel proximal to the reach of the colonoscope and also evaluate for extraluminal disease [8]. Perioperative assessment and management principles in CD are shown in Table 2 and recently reviewed [9].

Malnutrition is an independent risk factor for adverse postoperative outcomes and affects up to 70% of the IBD population [10, 11]. Malnourished patients undergoing surgery are at a particularly increased risk for postoperative complications given their systemic illness and the many other risk factors for poor surgical outcomes they may possess. This review will focus on perioperative nutritional support in IBD patients, with a particular emphasis on parenteral nutrition.

## 2. Surgical Techniques and Perioperative Medical Management

The cornerstone for elective surgical management of UC is a restorative total proctocolectomy with ileal pouch-anal anastomosis (IPAA). For patients with severe UC requiring urgent or emergent surgery who have been on high-dose steroids, a three-stage procedure is recommended with subtotal abdominal colectomy and end ileostomy performed initially, with a subsequent completion of proctectomy with IPAA [12]. In CD, surgery may be indicated for the treatment of refractory disease or for complications including strictures, obstruction, fistulae, abscesses, perforation, or dysplasia/cancer. Resection of a diseased segment of small or large bowel is often needed, with ileocecal resection being the most common surgery performed in CD. Alternatives to surgical resection such as stricturoplasty and occasionally endoscopic dilation are employed in the treatment of strictures in attempts to preserve bowel [13].

The optimal protocol for use of IBD therapies in the preoperative setting remains incompletely defined; however, several solid principles have emerged. Most importantly, steroids have a serious negative impact on postoperative outcomes [14]. In a meta-analysis by Subramanian et al. [15], patients on steroids have an increased risk of all postoperative complications (infectious and noninfectious) that was dose related. Zangenberg et al. [14] recommend a gradual withdrawal of steroids be attempted so that patients are steroid free for 1 week prior to surgery and recommend against adding additional “stress doses” of steroids for patients who remain on preoperative steroids. Some immunosuppressive medications, such as cyclosporine, are strongly associated with impairments in postoperative wound healing and therefore have higher rates of wound dehiscence, infections, and hernia formation [16]. The more commonly used thiopurines (azathioprine and 6-mercaptopurine) have minimal impact on postoperative complications [17, 18]. Studies investigating the impact of antitumor necrosis factors (anti-TNFs) on surgical outcomes have yielded mixed results, but more recent studies indicate that surgery can be safely performed on both CD and UC patients on biologic therapy [19, 20]. Further, preoperative anti-TNF therapy may have advantages such as decreasing operative times and decreasing the length of resection [21, 22].

Both the American Gastroenterological Association (AGA) and the American College of Gastroenterology (ACG) have published recent guidelines addressing medical therapy in the postoperative setting [23, 24]. While evidence is rapidly emerging, preoperative medical therapy remains institution or surgeon specific. These issues are particularly important to consider in CD because they can influence surgical approach and outcomes, complication rates, and postoperative disease recurrence rates. Addressing modifiable risk factors such as smoking and steroid use in CD is particularly important to achieve good outcomes [9, 14].

## 3. Identifying and Addressing Malnutrition in the Preoperative Period

Patients with active IBD are at a significantly increased risk for malnutrition, with prevalence rates ranging from 25 to 69% in this population [10]. Malnutrition can occur in UC but is a more common problem in CD since CD can affect any part of the GI tract and UC is restricted to the colon, which has few direct malabsorptive effects [25]. Depending on disease severity, 65% to 76% of CD patients experience weight loss in comparison to 18% to 62% of UC patients [26, 27]. Reasons for malnutrition in IBD patients are multiple and include: increased nutritional requirements from the associated hypermetabolic state seen in systemic inflammation, diarrhea, increased nutrient losses, reduced dietary intake, anorexia, drug-nutrient interactions, and nutrient malabsorption related to intestinal inflammation or reduced intestinal absorptive area from surgical resection [28, 29]. The European Society for Clinical Nutrition and Metabolism (ESPEN) therefore recommends that these patients be screened for malnutrition at the time of diagnosis and thereafter on a regular basis [30]. In addition, IBD patients, even those who appear well nourished, are vulnerable to micronutrient deficiencies (particularly vitamin D, iron, zinc, vitamin C, vitamin B12, and vitamin B6) and regular screening and deficit correction are recommended [28, 30, 31].

Defining malnutrition is difficult, especially in IBD patients, and a gold-standard test of malnutrition has not been identified [32]. Body mass index (BMI), unintentional weight loss, reduced dietary intake, nitrogen balance, and body fat percentage are commonly used clinical measures in the determination of nutritional status [28]. Wagner and Rombeau [32] arbitrarily defined malnutrition in perioperative patients as a serum albumin level of less than 3.5 g/dl and/or unintentional weight loss of 15% of a patient's usual weight over 3 to 4 months. In addition to serum albumin, prealbumin, transferrin, total cholesterol, and triiodothyronine (T3) have all been used as surrogate serologic markers to determine nutritional status but these markers are considered negative acute-phase reactants and are not specific for malnutrition, making them unreliable [32, 33]. Nevertheless, serum albumin of <3 g/dl is included as one of the best indicators of severe malnutrition in CD in the recent guidelines by ESPEN, in addition to a BMI < 18.5 kg/m^2^ and weight loss > 10–15% within six months [11, 20, 30, 34].

In hospitalized patients, malnutrition is an independent risk factor for venous thromboembolism, nonelective surgery, longer admission, and increased mortality [30]. Malnutrition is also a well-known risk factor for adverse postoperative outcomes in all surgical patients [11]. Up to 50% of patients with CD will require surgery at some point, and it is estimated that up to 85% of patients awaiting surgery are malnourished. Malnourished IBD patients may also possess several other risk factors for poor surgical outcomes. A recent meta-analysis by Huang et al. [35] identified steroid use, low albumin level, prior surgical history, and preoperative abscess (all common in CD) as risk factors for adverse surgical outcomes in CD patients. “Intra-abdominal septic complications” (anastomotic leaks, intra-abdominal abscesses, and enterocutaneous fistulae), one of the most dreaded complications in CD patients undergoing surgery, can occur in up to 20% of patients and frequently require reoperation or percutaneous drainage [11, 36]. Therefore, optimizing preoperative nutritional status in IBD patients is crucial for good surgical outcomes.

## 4. Preoperative Nutritional Support

### 4.1. Oral and Enteral Nutrition

In the preoperative period as in general, oral nutrition is preferred if patients can meet their energy and/or protein needs from normal food by mouth. The caloric need can be high due to malnutrition and the desire to enhance nutritional status before surgery. In addition, symptoms including nausea, abdominal pain, and diarrhea or high-volume fistula output can limit the ability to effectively utilize the gut. If patients are unable to meet their needs, oral nutritional supplements (ONS) that are high in protein and supplemented with vitamins and minerals can be added as a first step; however, they are generally a minor supportive therapy, providing a supplementary intake of only up to 600 kcal/day [30]. If caloric needs are not met through oral intake and/or oral feeding is not possible, enteral nutrition (EN) typically through a nasogastric or nasoenteric tube is indicated. Updated nutritional guidelines for patients with IBD were put forth by ESPEN in 2016 [30]. In these guidelines, most of the recommendations for preoperative nutritional support were based on consensus among experts or extrapolated from the considerable evidence of nutritional support in the general gastrointestinal surgical population and in critically ill patients [11].

Enteral nutrition is administered in either polymeric, semielemental, or elemental formulas depending on the presence of malabsorption [29]. The general consensus is that enteral nutrition (EN) is recommended for IBD patients undergoing surgery if they are able meet their metabolic demands, but if EN is not feasible (e.g., intestinal obstruction or high output fistula) or if metabolic needs are not met by EN alone, parenteral nutrition (PN) should be employed according to the established guidelines for use in the general population [11, 30, 37]. Compared to PN, EN carries decreased infectious risks, is less costly, and is more physiologic given that it promotes gastrointestinal growth and function [32, 38].

When EN is used to meet 100% of a patient's dietary requirements, it is termed exclusive enteral nutrition (EEN). There is an emerging body of literature demonstrating the role of EEN for primary therapy of Crohn's disease, particularly in the pediatric population. EEN has been shown to induce mucosal healing and have a direct anti-inflammatory effect by decreasing proinflammatory cytokines [11, 39]. In the pediatric CD population, EEN is the treatment of choice for induction of remission because it is as effective as corticosteroids in this regard (up to 80% of patients achieve remission) and sparing the detrimental side effects of corticosteroids (i.e., bone loss and growth retardation) [40, 41]. Studies demonstrating this effect in adult CD patients have been less conclusive due to issues with EEN compliance in this population [40].

Studies evaluating EEN use in the preoperative setting in CD patients have yielded promising results. Li et al. [42] demonstrated that preoperative EEN in patients with fistulizing CD is associated with a significantly decreased risk of intra-abdominal septic complications and may result in an accelerated recovery and ability to return to work sooner. A 2017 retrospective case-control study by Heerasing et al. [41] found that preoperative EEN frequently downstages the need for surgery in CD patients with stricturing or penetrating complications and is associated with reductions in systemic inflammation, operative times, and incidence of postoperative abscess or anastomotic leak. Wang et al. [43] observed that 4 weeks of preoperative EEN in CD patients led to significant reductions in both infectious and noninfectious complications and significant improvements in nutritional and inflammatory status. At 6 months postoperatively, significant reductions in endoscopic recurrence rates were seen in the EEN group; however, during the 2-year follow-up, this significance was lost. In addition, incidence of clinical recurrence was similar in the EEN and non-EEN groups during the 2-year follow-up [43]. A retrospective study of 83 patients by Zhu et al. [44] in 2017 found that for CD patients with percutaneously undrainable abscesses, the use of EEN was associated with a decreased need for surgery. In those patients who did require surgery, EEN was associated with increased albumin levels, decreased ESR and CRP levels, a lower risk of postoperative intra-abdominal septic complications, and a shorter duration of postoperative hospitalization compared to the group who did not receive EEN [44]. In a large retrospective 2015 study by Li et al. [45], the addition of EEN to a preoperative immunosuppressant-free interval led to lower rates of stoma creation, decreased postoperative complications, decreased urgent operation requirement, and extended preoperative immunosuppressant-free interval.

With regard to the optimal EN formula in CD, there are no significant differences between elemental, semielemental, and polymeric formulas in active CD [46]. Prior studies have shown that low-fat formulas may offer improved efficacy compared to formulas with high amounts of long-chain triglycerides but formulas with increased amounts of medium-chain triglycerides provided remission rates that were the same as those of low-fat formulas [47, 48]. A recent systematic review of 29 clinical trials [49] concurred that formulas with relatively low total long-chain triglyceride content and proportionately high medium-chain triglyceride content offer the most optimal approach for attaining remission in active CD. In addition, this study also concluded that ideal EN formulas were ones with relatively low amounts of monounsaturated fatty acids (MUFA) and high omega-6 : omega-3 fatty acid ratios [49].

In contrast to CD, there is no evidence that EEN alters the inflammatory response in UC, and therefore, it is not used in the treatment of active UC, nor in the maintenance of remission in UC [50]. However, according to ESPEN, EN has not been adequately evaluated in active UC and its efficacy needs to be tested in larger cohorts of patients [30]. EN can be used to treat or prevent malnutrition in UC patients and is safe to use as nutritional support during severe UC exacerbations and in the perioperative phase [30, 50].

There are certain clinical scenarios in which EN (or EEN) is not feasible. Poor tolerability of EN and poor palatability of formulations are major hindrances to patient compliance, and about 50% of patients relapse within 6 months and revert to a normal diet [39, 51]. In these cases, PN may be the next best alternative. PN is necessary when there is bowel obstruction and placement of a feeding tube beyond this obstruction is not possible. It is also required in patients with short bowel with severe malnutrition and/or fluid and electrolyte loss that is unable to be managed enterally [30]. High-output fistulae, inability to maintain enteral access, intractable nausea and vomiting, paralytic ileus, and inability to meet >60% of energy needs via the enteral route are also indications for PN [11, 29, 38].

### 4.2. Parenteral Nutrition

Only a few studies have examined the effect that perioperative PN has on postoperative outcomes.  Table 3 shows summaries and comparisons of these studies. Overall, the literature indicates that perioperative PN does not increase postoperative complications and that there is a general trend towards improvement in postoperative outcomes associated with perioperative PN use in IBD patients. However, the evidence supporting perioperative PN use is based on studies with small sample sizes that are limited with high degrees of heterogeneity with respect to study interventions, controls, measures, and outcomes. Therefore, as with preoperative medical therapy for CD, use of preoperative PN in this patient population is largely institution and surgeon specific.

Table 3 highlights details of the studies on the use of perioperative PN. Postoperative complications measured in these articles included: infections, perforation with free peritonitis, wound dehiscence, intestinal obstruction, fistulas, intra-abdominal abscess, anastomotic leak, intra-abdominal bleeding, and death. Of the seven studies reviewed in Table 3, two [33, 52] found statistically significant reductions in postoperative complications. Another study, the largest study to date to study perioperative PN in CD patients, found that preoperative PN for >60 days was associated with significantly lower odds of developing noninfectious complications (OR 0.07, 95% CI 0.01–0.80, *p* = 0.03) with no associated increase in infectious complications [20]. An additional study [53] did not find any significant differences in postoperative complications; however, statistical significances were observed with decreases in serum IgM (*p* = 0.04) and increases in BMI (*p* = 0.02) in the PN group when compared to the control group. One study [54] of exclusively UC patients saw more total postoperative complications in the PN group (50% vs 35.2% in the control group, *p* = 0.047); however, when line infections were taken out of the analysis, total complications were comparable (37.5% vs 35.2%, *p* = 0.753). The main outcome of interest in the study by Lashner et al. [55] was not postoperative complications, but complication rates were nearly identical in the two groups (49 total patients in PN groups with 10 complications, 54 total patients in control groups with 11 complications), indicating no increased risk of postoperative complications.

In general, measures of nutritional status and disease severity improved in patients receiving perioperative PN. More considerable BMI increases were noted with perioperative PN as compared with control groups in 2 studies [26, 53], with one observing statistical significance in this outcome [53]. Additionally, Jacobson [33] reported increases in BMI in the PN group but did not comment on BMIs in the control group. Jacobson [33] also stated that the PN group in their study had reductions in CDAI to the point of clinical remission by time of surgery; however, CDAI scores were not calculated in the control groups, and therefore, statistical significance could not be determined. Grivceva Stardevola et al. [26] saw that CDAI scores improve more in the PN group versus the control group but the results were not statistically significant.

Lashner et al. [52] found that preoperative PN was associated with reduced length of small bowel resection (albeit at the expense of a longer hospital stay). Since up to 70% of CD patients will require surgery at some point in their disease course, any interventions that can spare functional bowel and prevent short bowel syndrome are of the upmost importance. However, the referenced study is almost 30 years old and it does not appear that this specific measure has been studied in IBD patients receiving or not receiving PN preoperatively since then. Future studies should aim to measure the effect of perioperative PN on the length of bowel resection if possible in attempts to validate and possibly expand on the findings of this earlier study.

Regarding limitations, common themes were found among the studies and articles in this review. Small sample sizes were seen in all the studies. None of the studies controlled for diet, which is a significant limitation. Four of the seven research articles lacked PN composition data, which is also a significant limitation because it limits the ability to associate specific PN components with outcomes. Study designs were also understandably hindered for several reasons. Disease severity necessitating PN likely precluded the ability to randomize nutritional support in these patients. In addition, as shown by many of these studies, patients requiring PN are sicker at baseline, and therefore, directly comparing postoperative outcomes in this subset of patients to outcomes of control groups would be inherently flawed. Only a small number of studies used propensity matching or other statistical techniques to control for disease severity and other differences between groups. Also, blinding is obviously difficult when comparing PN to EN or oral nutritional intake.

PN can be given through peripheral lines, but since either increased fluid provision or limited nutrient content in the solution to maintain an osmolarity ≤ 900 mOsm/l is needed, it is usually given through a central venous catheter [28]. Central venous catheters have historically carried a higher risk of bloodstream infections, which has led to significant healthcare costs as well as patient morbidity and mortality. In a 2005 study, Beghetto et al. [56] asserted that PN was an independent risk factor for central venous catheter-related infection in nonselected hospitalized adult patients. PN has been associated with noninfectious complications as well, such as refeeding syndrome, hyperglycemia, hypertriglyceridemia, hepatic steatosis, intrahepatic cholestasis, biliary sludge, and bone demineralization [57]. However, as Cotogni [58] points out, the incidence of PN-related complications has markedly decreased in recent years due to proven, standardized policies that reduce catheter-related bloodstream infections. In addition, other provisions including appropriate blood glucose control, use of olive oil- and fish oil-based lipid emulsions rather than soybean oil-based ones, and the adoption of insertion and care bundles for central venous access devices have moved the field closer to the goal of “near-zero” PN complications [58, 59]. Also, there is increased awareness of the association between overfeeding and infectious complications [60]. Two recent randomized controlled trials (the CALORIES trial [61] and NUTRIREA-2 [62]) studying early nutritional support in critically ill patients found no statistically significant differences in infectious complications between the EN and PN groups. In the 2018 study by Ayoub et al. from our group at the University of Florida [20], there were no statistical differences seen in infectious complications between preoperative IBD patients on PN and control patients (18.2% in the PN group, 12.3% in the non-PN group, *p* = 0.34). In our study, a subset of malnourished patients benefited from PN in the preoperative period. Our findings concur with Schwartz [28] who proposed that IBD patients with underlying severe malnutrition may be the better candidates for perioperative PN since the benefit of preoperative nitrogen balance optimization may outweigh any infectious risks incurred by placing a central venous catheter for PN.

Omega-3 fatty acids (contained in fish oil) have anti-inflammatory activity via their ability to regulate inflammatory processes and cellular responses [63]. Given this, its use in IBD has warranted investigation. In a rat model of experimental colitis, omega-3-enriched parenteral lipid emulsions decreased colonic concentrations of proinflammatory mediators and attenuated the inflammatory consequences of colitis [63]. The ICU lipid study [64] showed that parenteral administration of a lipid emulsion containing 10% fish oil reduced the risk of nosocomial infections in critically ill medical and surgical patients, although it did not have an effect on ICU, hospital, or 6-month mortality. While two recent Cochrane reviews [65, 66] failed to show any evidence to support the use of oral omega-3 fatty acids for maintenance of remission in CD or UC, studies investigating the use of omega-3-enriched parenteral lipid emulsions in the human IBD population are lacking. Further studies are needed before fish oil in PN can be routinely recommended in the IBD population.

Home PN is sometimes indicated in select patients with CD, such as those with chronic intestinal failure from short bowel syndrome (which is usually a consequence of repeated small bowel resections), prolonged incomplete bowel obstruction, high-output stomas, and persistent fistulas [25, 27]. Home PN has not been associated with significant increases in body weight or reductions in the frequency of further CD-related surgeries. However, increased quality of life, higher serum albumin and transferrin levels, and a reduction in oral steroid requirements have been noted [25]. Complications of home PN are the same as those of hospital-administered PN and include catheter-related infections, cholestasis, refeeding syndrome, and bone demineralization [27]. While the benefits of perioperative PN illustrated in this review could theoretically be applied to home perioperative PN use, home PN use in the perioperative period has not been studied, and therefore, there is insufficient evidence either for or against its use.

## 5. Preoperative Optimization

Optimizing nutritional status preoperatively is crucial to mitigating postoperative complications. According to ESPEN guidelines [30] published in 2017, surgical intervention in IBD patients should ideally be delayed for 7–14 days whenever possible if malnutrition is identified, during which time-intensive artificial feeding (EN preferably) should be initiated [30]. When surgery cannot be delayed or emergency surgery is needed, ESPEN [30] recommends that nutritional support (preferably EN over PN if possible) be initiated without delay in patients who are malnourished and even in patients without significant malnutrition if it is expected that the patient will be unable to eat for more than seven days perioperatively. Nutritional support should also be provided in patients who cannot maintain oral intake above 60–75% of recommended intake for more than 10 days. In cases where EN is initiated in hospitalized patients, additional supplemental PN should be introduced if after 7–10 days of EN, the patient is unable to meet >60% of energy and/or protein requirements. There is evidence that adding supplemental PN prior to this 7–10-day period in those patients already receiving EN does not improve outcomes and may actually be detrimental [67].

The timing of surgery in patients with malnutrition deserves additional comment. While the optimal duration for preoperative PN is not certain, one could argue that a surgical delay of 7–14 days cited by ESPEN [30] is not long enough to optimize IBD patients preoperatively with PN. Patel et al. [9] continue PN until albumin levels are >3 g/dl, CRP levels are <5 mg/l, and BMI improves; in the only study reporting these improved outcomes [33], this approach took a minimum of 18 days and a mean of 46 days of PN treatment. Our group [20] showed that PN for >60 days preoperatively in CD patients was associated with lower odds of developing noninfectious complications with no associated increase in infectious complications. To our knowledge, the optimal duration of preoperative PN in UC patients has not been studied. Clearly, a balance of contributing factors exists, and, in the absence of convincing evidence to guide decision making, this remains institution specific with good opportunities for additional investigation. Since universal recommendations favor the use of EN over PN whenever possible, it is not surprising that patients in need of PN are those with the most complicated and severe disease [30]. This was corroborated in several studies in this review [20, 26, 33, 52, 54] showing that patients requiring PN were more malnourished with higher disease activity and lower albumin levels at baseline compared to control groups. For this reason, all possible steps to improve these patients' preoperative conditions should be taken. The concept of preoperative optimization has shown promising data in IBD [11, 14, 68, 69]. This concept is based on a multimodal approach (including nutritional strategies) to optimize a patient's preoperative condition and to reduce the risk of unfavorable postoperative outcomes.

Studies on preoperative optimization have demonstrated favorable outcomes. Zerbib et al. [69] reported retrospectively on the results of preoperative optimization for patients undergoing ileocecal resection for penetrating CD, with focus on rates of major postoperative complications and need for fecal diversion. 50 of the 74 total patients (64%) received two weeks of preoperative nutritional support with either EN (5 patients) or PN (45 patients) in combination with bowel rest, two weeks of intravenous antibiotics, withdrawal from steroids and immunosuppressants, and/or abscess drainage. The postoperative morbidity rate was low (18%), with only six temporary fecal diversions and no deaths. Similarly, Bellolio et al. [68] compared surgical outcomes of CD patients with perforating versus nonperforating disease following ileocolic resection. In this study of 434 patients, 56 received preoperative PN because of septic complications or malnourishment. Patients with perforating disease were more likely to be the ones receiving preoperative PN (17.1% vs 4.3%, *p* < 0.001). They found that PN in combination with antibiotics, abscess drainage, and postponed surgery in patients with perforating disease led to similar complication rates compared with that in patients with nonperforating disease (13% vs 11%). A systematic review of 50 studies by Zangenberg et al. [14] examined preoperative optimization in IBD patients undergoing gastrointestinal surgery and recommended weaning of steroids if possible, avoidance of stress dose of steroids, percutaneous drainage of abscesses if present, smoking cessation, appropriate thromboembolism prophylaxis, administration of prophylactic IV antibiotics, and screening for malnutrition with appropriate nutritional support. They concluded, however, that large prospective studies comparing different types and duration of preoperative nutrition are needed, especially in UC patients since most studies deal with CD.

## 6. Conclusion

Addressing nutritional needs of IBD patients in the perioperative period can improve patient outcomes. Nutritional assessment and perioperative nutritional support are often neglected components of good disease management. When providing nutritional support, the enteral route is favored over the parenteral route in most cases. In certain severe IBD cases, PN seems to reduce postoperative complications, improve nutritional status, and reduce disease severity. Our recommendation is that the current use of perioperative PN in IBD patients should be in line with the ESPEN guidelines for PN in the general surgical population [37]. That is, PN should be considered preoperatively only if metabolic needs are not met by EN alone or if disease presentation at the time of surgery impedes the use of EN (e.g., high-output fistula or intestinal obstruction) [11]. Additional, well-controlled studies of EN and PN in the perioperative setting are warranted.

## Figures and Tables

**Table 1:** Predictors of aggressive Crohn's disease [4–7].

(i) Clinical risk factors
(a) Young age at presentation
(b) Steroids required at first presentation or within 6 months
(c) Perianal disease
(d) Upper tract disease
(e) >2 steroid courses
(f) Current smokers
(g) Multiple admissions
(h) Early resection
(ii) Increased number of positive antibodies identified children at risk for complicated disease
(a) ANCA, ASCA, and anti-CBir 1
(iii) One or more NOD2 mutations associated with aggressive fibrostenotic course
(iv) Presence of a stricture on CTE, MRE, or colonoscopy risk factor for future complications (fistula, abscess, perforation, and obstruction)

ANCA: antineutrophil cytoplasmic antibodies; ASCA: anti-Saccharomyces cerevisiae antibodies; anti-CBir 1: bacterial flagellin antibodies; NOD2: nucleotide-binding oligomerization domain-containing protein 2; CTE: computed tomography enterography; MRE: magnetic resonance enterography.

**Table 2:** Preoperative assessment and management in CD patients [9].

(i) Preoperative cross-sectional imaging
(a) Identify sites of inflammation and assess for abscess, fistula, and stricture
(ii) Address modifiable risk factors
(a) Smoking—stop preoperatively, even 4 weeks prior shows benefit
(b) Steroids—wean preoperatively (ideally 4 weeks preoperatively)
(c) Anemia—IV iron often needed, transfusion not usually indicated
(iii) Optimize nutritional status when indicated
(a) Weight loss > 10–15% within 6 months
(b) Body mass index < 18.5 kg/m^2^
(c) Serum albumin < 3 g/dl (with no evidence of hepatic or renal dysfunction)
(iv) Preoperative colonoscopy (typically but not uniformly needed)
(v) Medical therapy through surgery
(a) Thiopurines—no change
(b) Anti-TNFs—assess levels and antibodies, try not to interrupt
(c) Other biologics—little or no data
(d) Thromboembolism prophylaxis—inpatient

IV: intravenous; anti-TNF: tumor necrosis factor inhibitors.

**Table 3:** Characteristics of primary studies investigating the use of perioperative PN in IBD patients.

Author and year	Patient population and interventions	Measured outcomes	Results	Conclusions	Study strengths	Study limitations
Rombeau et al. [52] 1982 retrospective cohort study	Patients with IBD undergoing abdominal surgery; group 1: 11 patients with 0–5 days preoperative PN versus group 2: 22 patients with ≤5 days of preoperative PN	Postoperative complications and length of hospital stay	(i) Group 2 (≤5 days preoperative PN) had significantly fewer postoperative complications (*p* < 0.05); no statistically significant difference in length of hospital stay (ii) All patients with postoperative complications had either a preoperative serum albumin level <3.5 g/dl or a serum transferrin level < 150 mg/dl	Preoperative PN for at least 5 days is strongly recommended in patients with IBD who have severe protein deficiency	(i) Study groups comparable with respect to demographic data, diagnoses, and types of surgery (ii) Preoperative nutritional status was comparable in the two groups	(i) Small sample size (ii) Lack of PN composition data (iii) Lack of control for diet in group 1 patients who did not receive PN

Lashner et al. [55] 1989 retrospective cohort study	103 patients with CD undergoing bowel resection (segmental small bowel resection, ileocecectomy, or segmental or total colectomy). Preoperative PN vs control groups compared between surgery types	Length of resected bowel and length of hospital stay	(i) Preoperative PN associated with reduced length of small bowel resection (20.4 ± 14.3 cm less bowel resected in those undergoing segmental small bowel resection and 11.2 ± 4.2 cm less in those undergoing ileocecectomy) that was independent of length of disease determined preoperatively (ii) No difference in length of large bowel resected between the PN and non-PN groups (iii) PN associated with a longer hospitalization (13.5 ± 2.6 days longer for the PN group)	Preoperative PN for CD patients is beneficial for those undergoing small bowel resection but was of little benefit for those undergoing colectomy	Controlling for confounding variables with the multivariate regression model did not change the results	(i) Lack of PN composition data (ii) Lack of control for diet in non-PN groups (iii) Patient selection bias (criteria for patient selection varied among attending physicians and no standardized criteria were established)

Yao et al. [53] 2005 nonrandomized controlled trial	32 severely malnourished CD patients (BMI < 15.0 kg/m^2^) undergoing abdominal surgery; 16 patients received 1 week of preoperative PN and 3 weeks of postoperative PN versus 16 patients in the control group received intravenous fluids with an isocaloric diet “comparable in energy to PN”	Serum immunoglobulins, BMI, weight change, liver function, postoperative complications, and return to work	(i) No significant differences in postoperative complications between groups (ii) BMI significantly increased in the PN group (from 13.9 ± 0.6 to 15.3 ± 0.7 kg/m^2^, *p* = 0.02) and did not change significantly in the control group (14.1 ± 0.7 to 14.5 ± 0.5 kg/m^2^, *p* = 0.81) (iii) Serum IgM levels in the PN group declined to normal value (139 ± 41 to 105 ± 29 mg/dl, *p* = 0.04), whereas no statistically significant change is seen in control group's IgM levels (from 133 ± 16 to 129 ± 13 mg/dl, *p* = 0.34) (iv) More patients in the study group returned to work than in the control group	Perioperative PN possibly ameliorates the humoral immunity, reverses malnutrition, and facilitates rehabilitation	(i) Novel study measures able to show objective improvement in nutritional status and humoral immunity with preoperative PN (ii) Equal number of patients in the study vs control groups (important in studies with small number of patients)	(i) Small sample size (ii) Limited description of results (iii) Methods for controlling for diet were not specified

Grivceva Stardelova et al. [26] 2008 retrospective/prospective cohort study	90 patients with IBD undergoing abdominal surgery; 29 patients (16 CD, 13 UC) received an average duration of 19.1 ± 18.5 days of preoperative PN and 61 patients (50 CD, 11 UC) did not receive PN	BMI, disease activity index (CDAI/AI), laboratory indices, and length of hospital stay	(i) CDAI scores decreased in the PN group more than in the control group and BMI increased more in the PN group than in the control group; however, neither of these findings was statistically significant (no *p* values provided) (ii) No statistically significant difference in length of hospital stay	Patients in the PN group had more severe baseline disease (lower BMIs and higher CDAI scores of statistical significance). Although improvements in BMI and CDAI are not statistically significant, PN aided improvement in clinical status to the level of the healthier controls. Authors estimate that duration of effective PN should be at least 7–14 days	(i) First study that investigated the effect of PN on disease activity scores	(i) Relatively small sample size (ii) Lack of PN composition data (iii) Lack of control for diet in the non-PN group (iv) Inconsistent reporting of statistical significances and vague explanations of the significant differences in laboratory indices

Jacobson [33] 2012 nonrandomized controlled trial	120 patients with moderate to severe CD undergoing intestinal resection. 15 patients received preoperative PN versus 105 patients not given PN preoperatively	Early (within 30 days) postoperative complications	(i) A significant reduction in early postoperative complications (*p* < 0.05) was seen in the PN group (0 patients with complications) versus the control group (29 patients with complications) (ii) Results suggested that preoperative PN increased body weight, serum albumin, and serum triiodothyronine, which reflected an improved nutritional state as well as a lowered risk of postoperative complications. (iii) PN also decreased serum haptoglobin and white blood cell count which suggested decreased inflammatory activity	Patients with moderate to severe CD undergoing intestinal resection should be treated with at least 18 days of preoperative PN to lower the risk of early surgical complications	(i) PN solution composition provided (ii) Lab markers for nutrition and inflammation were measured, and authors were able to show that improvements in these measures were associated with preoperative PN use	(i) Small sample size (ii) Lack of control for nutrient intake in the control group (iii) Average CDAI for control subjects was not calculated (iv) Sevenfold matching based on baseline characteristics (v) Controls were historical rather than contemporaneous (vi) PN compositions varied widely

Salinas et al. [54] 2012 retrospective cohort study	235 UC patients undergoing abdominal surgery. 56 patients received preoperative PN and 179 did not	Early (within 30 days) postoperative complications	(i) PN was associated with more total postoperative complications (50% vs 35.2% in the control group, *p* = 0.047); however, when line infections were excluded, total complications were comparable (37.5% vs 35.2%, *p* = 0.753)	Routine preoperative PN for the general population of UC patients undergoing surgeries is not indicated	(i) Larger sample size compared to other studies (ii) Total and individual postoperative complications were compared between groups	(i) Statistically significant disparities in baseline nutritional status and disease severity between groups (ii) Lack of control for diet in the non-PN group (iii) Lack of PN duration and composition

Ayoub et al. [20] 2018 retrospective cohort study	144 CD patients undergoing major abdominal surgery; 55 received preoperative PN and 89 did not	30-day postoperative complications	(i) No statistical differences were seen in infectious complications (18.2% in the PN group, 12.3% in the non-PN group, *p* = 0.34) or noninfectious complications (14.5% in the PN group, 16.8% in the non-PN group, *p* = 0.71) (ii) Patients receiving preoperative PN for >60 days had significantly lower odds of developing noninfectious complications (OR 0.07, 95% CI 0.01–0.80, *p* = 0.03) with no associated increase in infectious complications (iii) Weight loss of >10% in the past 6 months was a significant predictor of postoperative complications	In a subset of malnourished CD patients, PN is safe and allows comparable postoperative outcomes to controls	(i) Largest study to date on preoperative PN in CD patients (ii) Detailed description and comparison of baseline patient characteristics, including disease and surgical characteristics between groups (iii) Authors provided a detailed description of rationale for initiation of PN	(i) Lack of a validated preoperative nutritional assessment for all patients

PN: parenteral nutrition; IBD: inflammatory bowel disease; CD: Crohn's disease; BMI: body mass index; UC: ulcerative colitis; CDAI: Crohn's disease activity index.
